# Supplementary material for: A fungal phospholipase C involved in the degradation of plant glycosylinositol phosphorylceramides during Arabidopsis/Botrytis interaction
Source: Commun Biol. 2024 Oct 22;7:1372. doi: 10.1038/s42003-024-07064-x (PMC11496612; doi:10.1038/s42003-024-07064-x)
Supplement: Supplementary file 2 — Supplementary Information [file 42003_2024_7064_MOESM2_ESM.pdf]

Supplementary Materials for

A fungal phospholipase C in the degradation of plant glycosylinositol phosphorylceramides during Arabidopsis/Botrytis interaction

Luka Lelas, Justine Rouffet, Alexis Filachet, Julien Sechet, Antoine Davière, Thierry Desprez, Samantha Vernhettes, Aline Voxeur

Corresponding authors: aline.voxeur@inrae.fr; samantha.vernhettes@inrae.fr

The PDF file includes:

Materials and Methods
Supplementary Text
Figs. S1 to S7
Table S1

Other Supplementary Materials for this manuscript include the following:

Data S1

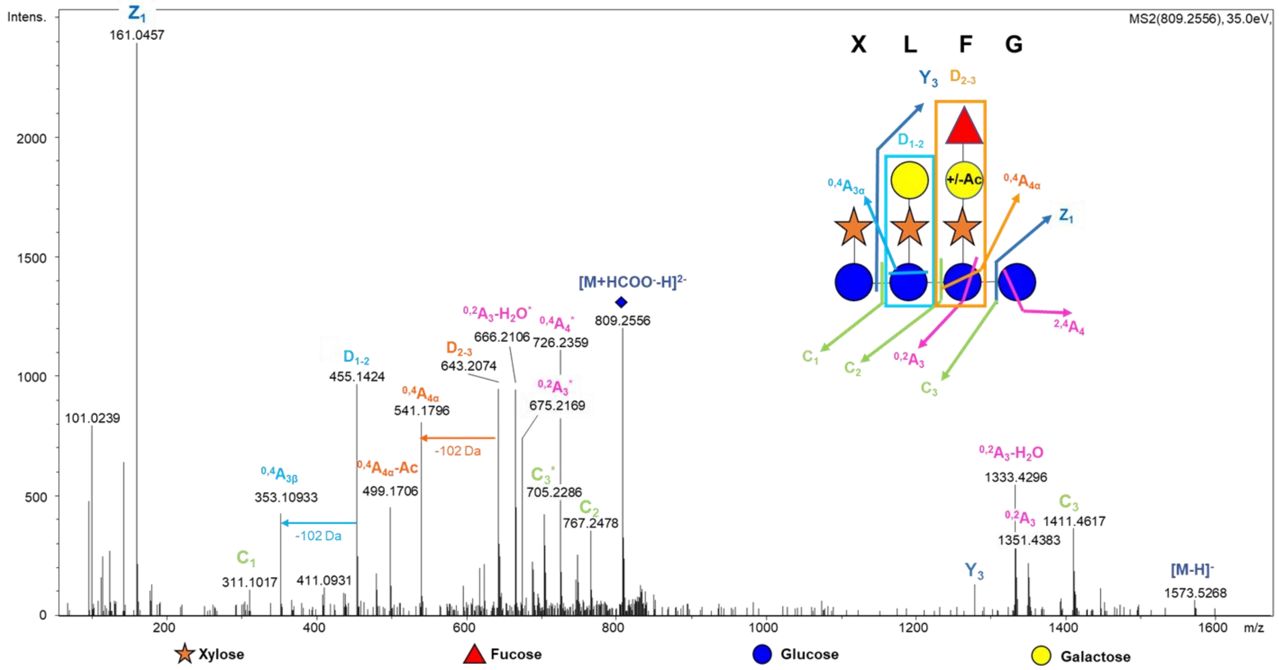

Fig. S1. An acetylated and fucosylated xyloglucan oligosaccharide is accumulated upon *Arabidopsis thaliana-Botrytis cinerea* infection. MS^2 fragmentation pattern of *m/z* 809 and a proposed fragmentation scheme.

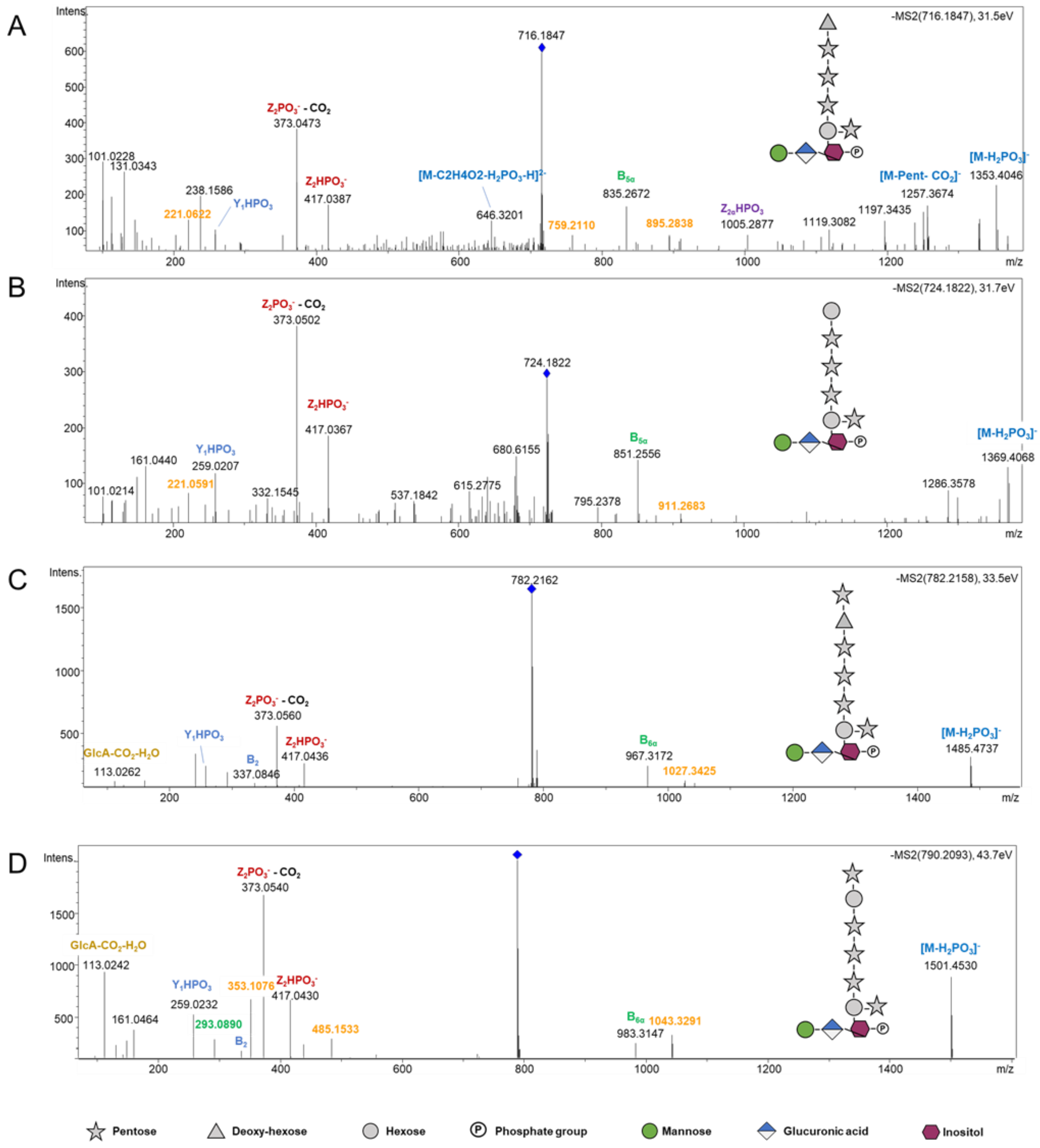

Fig. S2. Inositol (Phosphate) Glycans (I(P)Gs) accumulate during *Arabidopsis thaliana*-*Botrytis cinerea* infection MS^2 fragmentation pattern of *m/z* (**A**) 716, (**B**) 724, (**C**) 782 and (**D**) 790 in negative mode. GlcA: Glucuronic acid, Intens.: signal intensity.

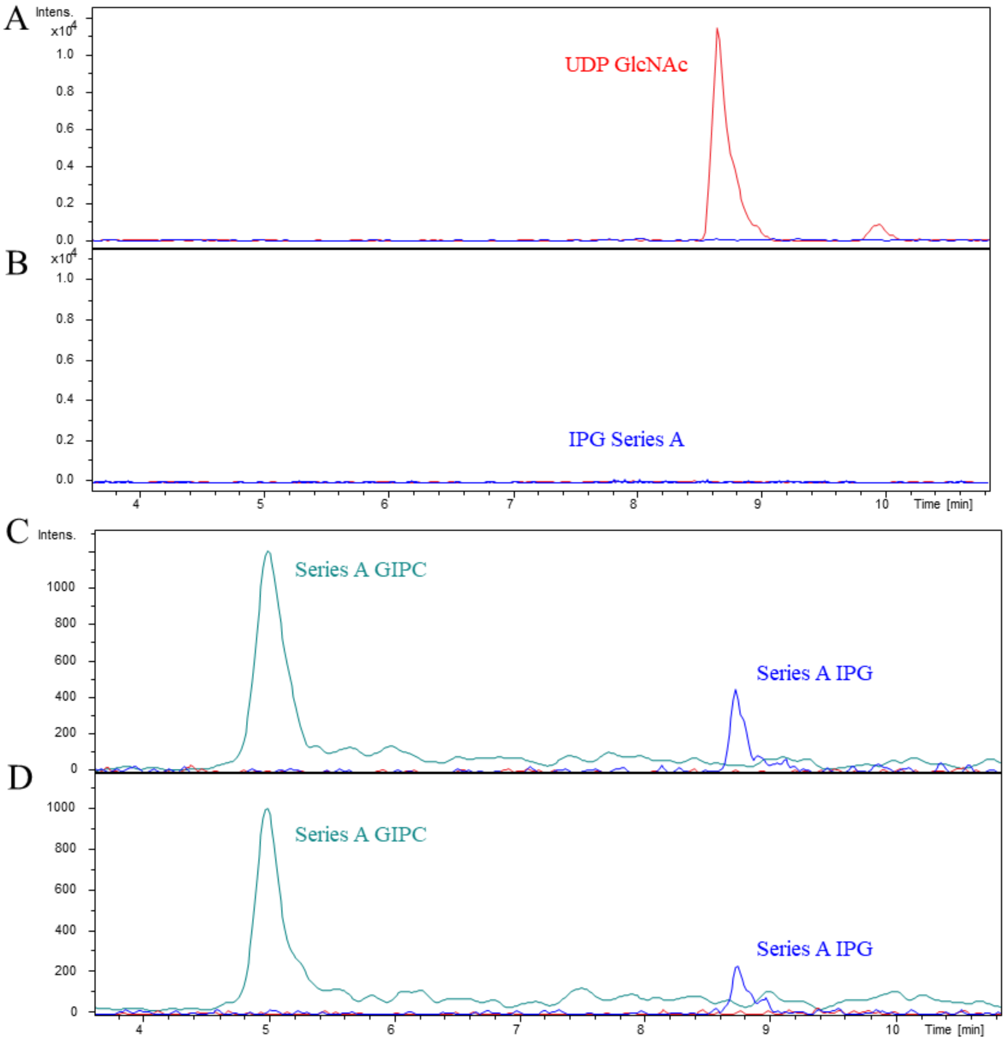

Figure S3. **BcWT grows on pectins but not on GIPC as monitored by the production of uridine diphosphate N-acetylglucosamine (UDP-GlcNAc).** (**A**) Extracted ion chromatograms of accumulated UDP-GlcNAc (red) and Series A IPG (blue) over time. *B. cinerea* conidia were incubated overnight with commercial pectins. The production of Series A IPG and UDP-GlcNAc was monitored using HPSEC-HRMS. (**B**) Extracted ion chromatograms of UDP-GlcNAc (red) and Series A IPG from commercial pectins not incubated with fungal conidia. (**C**) Extracted ion chromatograms of Series A GIPC t18:1 h22:0 (green), Series A IPG (blue) and UDP-GlcNAc (red) prepared from incubating *A. thaliana* GIPC-enriched extract with *B. cinerea* conidia in a liquid culture medium overnight. (**D**) Extracted ion chromatograms of Series A GIPC t18:1 h22:0 (green), Series A IPG (blue) and UDP-GlcNAc (red) prepared from incubating *A. thaliana* GIPC-enriched extract in a liquid culture medium not containing fungal conidia overnight.

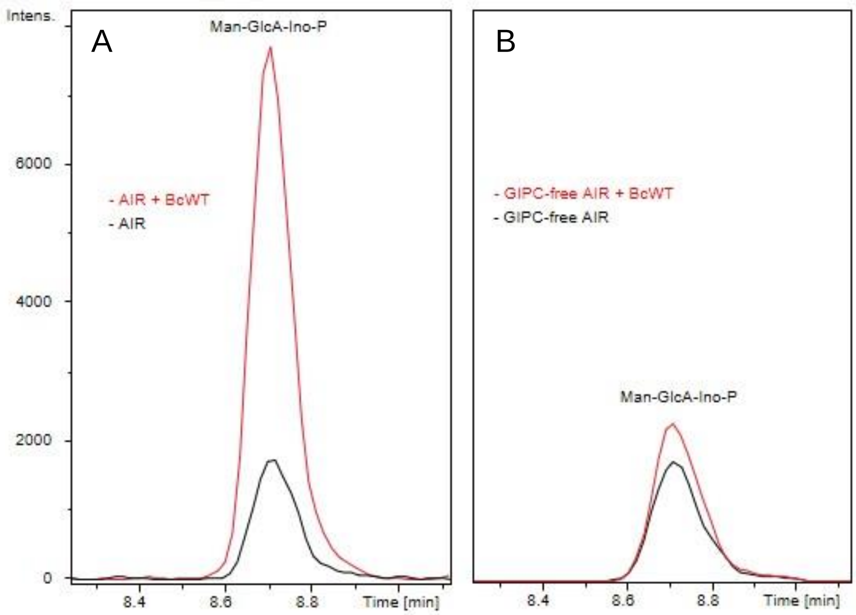

Figure S4. **BcWT does not produce IPGs from GIPC-free AIR.** (**A**) Extracted ion chromatograms of accumulated Series A IPG. AIR was incubated overnight with water (black) or *B. cinerea* conidia (red) and the production of Series A IPG was monitored using HPSEC-HRMS. (**B**) Extracted ion chromatograms of accumulated Series A IPG. GIPC-free AIR was incubated overnight with water (black) or *B. cinerea* conidia (red) and the production of Series A IPG was monitored using HPSEC-HRMS.

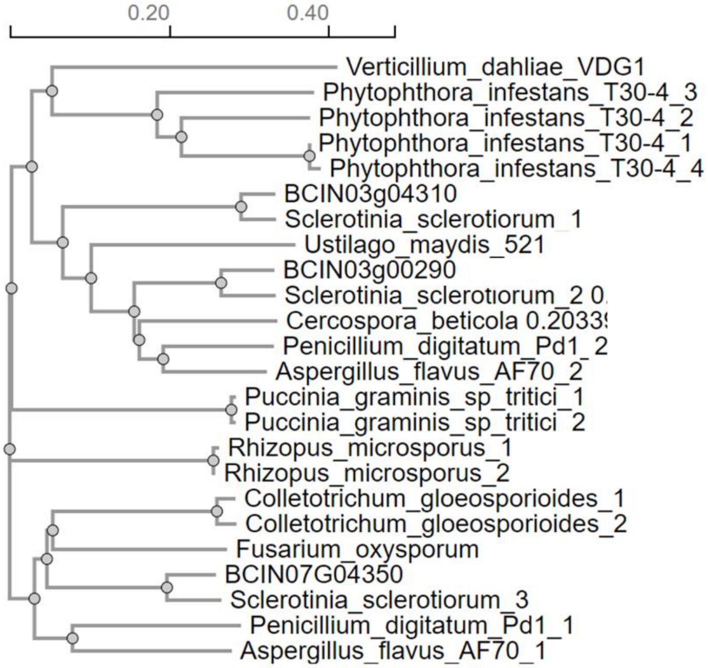

Figure S5: **Phylogenetic tree of *B. cinerea's* sphingomyelinase encoding genes and related phytopathogen fungi genes**. The tree depicts the evolutionary relationships among various fungal sphingomyelinase encoding genes with branch lengths representing genetic distances. Sphingomyelinase encoding genes are divided into two clades: the upper clade includes *Penicillium digitatum* (necrotrophic and saprotrophic), *Aspergillus flavus* (saprotrophic), *Cercospora beticola* (hemibiotrophic), and *Sclerotinia sclerotiorum* (necrotrophic), along with genes *BCIN03g00290* and *BCIN03g04310*. The lower clade includes *Puccinia graminis* (biotrophic), *Colletotrichum gloeosporioides* (hemibiotrophic), *Fusarium oxysporum* (necrotrophic and hemibiotrophic), and BCIN07G04350. Finally, the middle clade primarily comprised various variants of *Phytophthora infestans* (hemibiotrophic) and *Verticillium dahlia* (hemibiotrophic). This suggests the widespread conservation of sphingomyelinase encoding genes proteins among major fungal lineages, independently of their lifestyle, with a notable exception in the genus Alternaria. Phylogenetic analysis was performed using ClustalW (default settings).

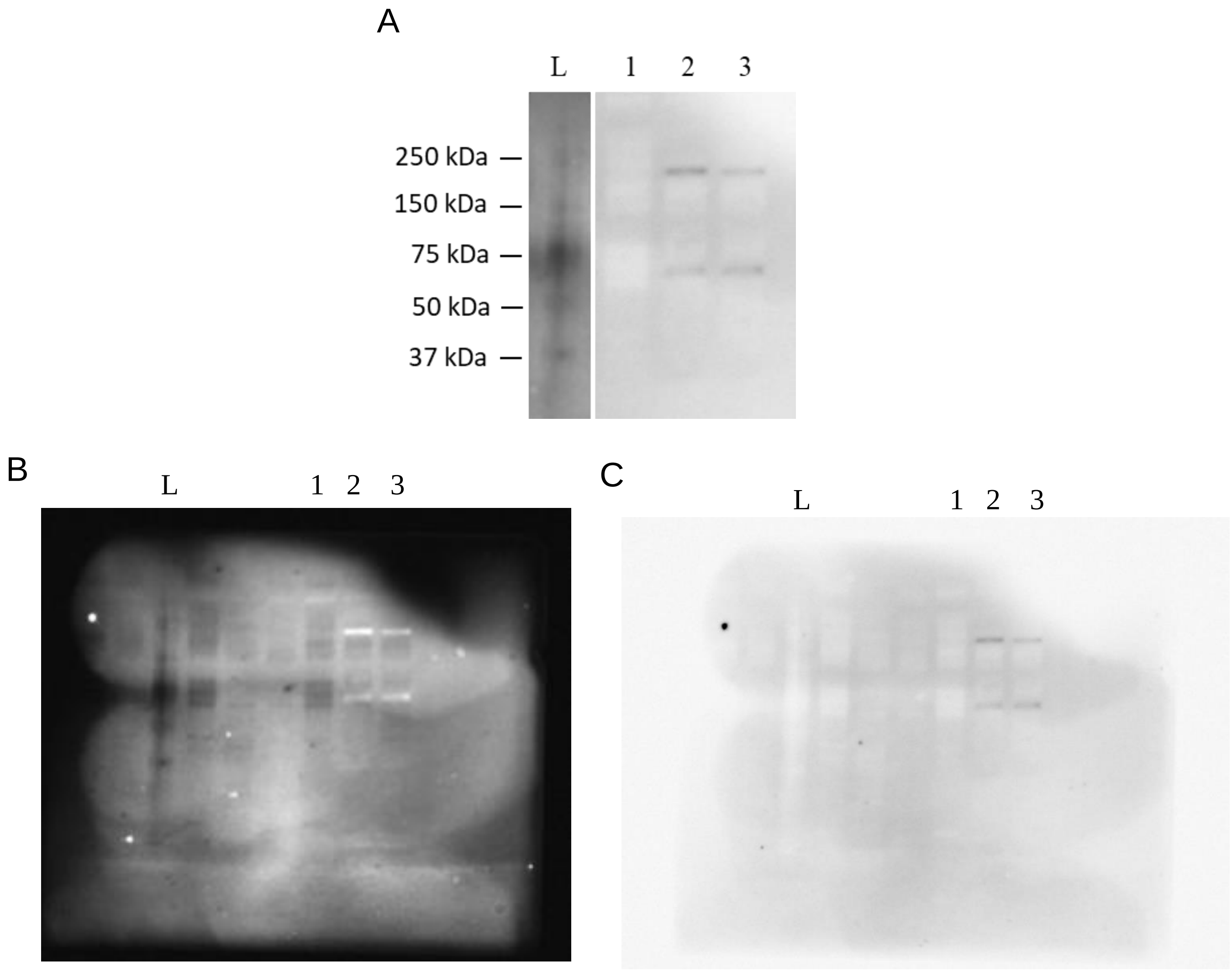

Fig. S6. Secretion of the BcGIPC-PLC (68.5 kDa expected weight) encoded by *BCIN_07g04350* in the X33 *Pichia pastoris* strain. A. Anti-His antibodies and chromogenic detection were employed. L: molecular weight markers with an acquisition time of 17 min in negative mode. 1. Proteins extracts from the supernatant of non-induced *BcGIPC-PLC* transformant culture. 2. Protein extracts from the supernatant of *BcGIPC-PLC* transformant culture n°1 induced with methanol to express the transgene during a period of 72 h. 3. Protein extracts from the supernatant of *BcGIPC-PLC* transformant culture n°2 induced with methanol during 72 h. Two-minute-long acquisitions were used to reveal the His-tagged expressed proteins. **B.** Corresponding uncropped and unedited of chromogenic detection with an acquisition time of 17 min. **C**. Corresponding uncropped and unedited of chromogenic detection in negative mode with an acquisition time of 2 min.

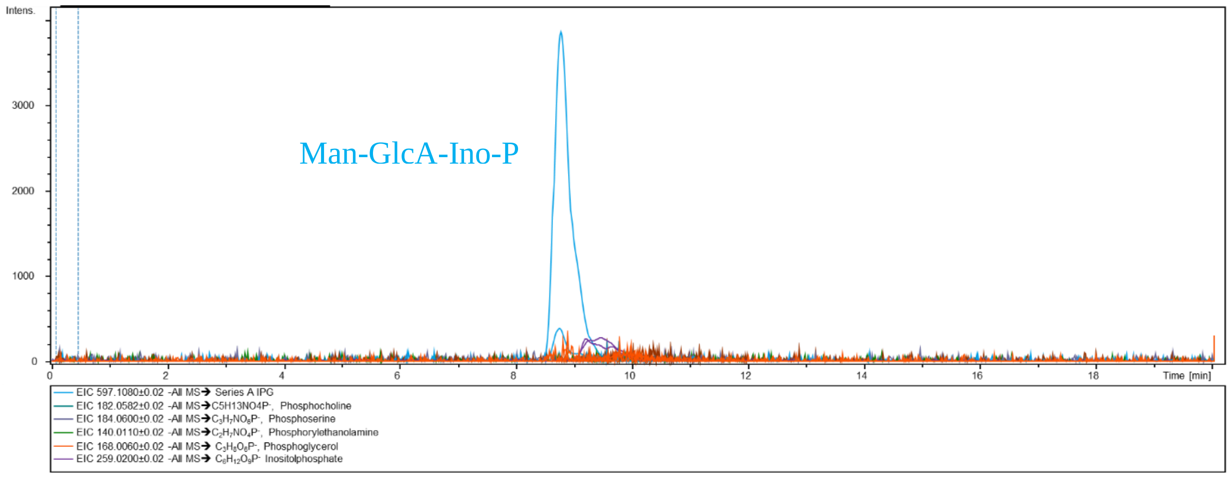

Fig. S7. The BcGIPC-PLC specifically degrades plant GIPCs. Extracted ion chromatograms of accumulated Series A IPG (m/z 597.10), phosphocholine (m/z 182.06), phosphoserine (m/z 184.06), phosphorylethanolamine (m/z 140.01), phosphoglycerol (m/z 168.01) and inositolphosphate (m/z 259.02) over time. BcGIPC-PLC was incubated over night with *A. thaliana* AIR that contains both cell wall and plasma membrane and the production of each polar head was monitored using HPSEC-HRMS.

Table S1.

Gene ID	Spearman's rank correlation coefficiency	P-Value
**BcPG1**	**0,472011904**	**0,000**
**Bcin07g04350.1**	**0,310575234**	**0,000**
**Bcin03g00290.1**	**0,191111165**	**0,005**
**Bcin03g04310.1**	-0,008587725	0,924

Table S1: Spearman's rank correlation coefficient between *A. thaliana* lesion area and *B. cinerea* SPHINGOMYELINASE transcript accumulation, as obtained from Zhang et al. (2019). BcPG1 was included as a reference.

Data S1. (separate file)

Retention time, mass-to-charge ratio and peak area of candidates.
