# Supplementary material for: A fungal phospholipase C involved in the degradation of plant glycosylinositol phosphorylceramides during Arabidopsis/Botrytis interaction
Source: Commun Biol. 2024 Oct 22;7:1372. doi: 10.1038/s42003-024-07064-x (PMC11496612; doi:10.1038/s42003-024-07064-x)
Supplement: Supplementary file 3 — Description of Additional Supplementary Files [file 42003_2024_7064_MOESM3_ESM.pdf]

Description of Additional Supplementary Files

File name: Supplementary data 1
Description: Retention time, mass-to-charge ratio and peak area of candidates.
